# Green Myco-Synthesis of Zinc Oxide Nanoparticles Using *Cortinarius* sp.: Hepatoprotective, Antimicrobial, and Antioxidant Potential for Biomedical Applications

**DOI:** 10.3390/microorganisms13050956

**Published:** 2025-04-22

**Authors:** Uzma Fazal, Ahmad Zada, Muhammad Hanif, Shiou Yih Lee, Mohammad Faisal, Abdulrahman A. Alatar, Tahira Sultana, Sohail

**Affiliations:** 1 College of Bioscience and Biotechnology, Yangzhou University, Yangzhou 225009, China; fazaluzma15@gmail.com (U.F.); sohail.botanist@hotmail.com (S.); 2 Department of Botany, Government College University, Lahore 54000, Pakistan; 3 Faculty of Health and Life Sciences, INTI International University, Nilai 71800, Negeri Sembilan, Malaysia; shiouyih.lee@newinti.edu.my; 4 Department of Botany & Microbiology, College of Science, King Saud University, P.O. Box 2455, Riyadh 11451, Saudi Arabia; faisalm15@yahoo.com (M.F.); aalatar@ksu.edu.sa (A.A.A.); 5 Department of Botany, PMAS Arid Agriculture University, Rawalpindi 46300, Pakistan; tahirasultana95@gmail.com

**Keywords:** *Cortinarius* sp., ZnO-MNPs, anti-cancer, antimicrobial, antioxidant

## Abstract

The transformative effect of nanotechnology is revolutionizing medicine by introducing new therapeutic approaches. This study explores the utilization of aqueous extract from mushroom (*Cortinarius* sp.) used as a reducing agent to prepare zinc oxide myco-nanoparticles (ZnO-MNPs) in an eco-friendly manner. The synthesis of ZnO-MNPs has been confirmed by various characterization studies, including UV-vis spectroscopy, which revealed an absorption peak at 378 nm; X-ray diffraction (XRD) analysis, which revealed a wurtzite hexagonal structure; and Fourier transform infrared spectra (FTIR), which showed stabilizing agents around the ZnO-MNPs. The effectiveness of ZnO-MNPs as an anti-cancer agent was evaluated by monitoring liver biochemical parameters against hepatotoxicity caused by carbon tetrachloride (CCl_4_) in Balb C mice. The results showed that the levels of catalase, glutathione (GSH), and total protein were significantly lower, while alanine aminotransferase (ALT), aspartate aminotransferase (ASAT), alkaline phosphatase (ALP), lactate dehydrogenase (LDH), melanin dialdehyde (MDA), and total bilirubin (TB) were significantly higher in each of the CCl_4_ treatment groups. ZnO-MNP treatment significantly reduced the toxicological effects of CCl_4_ but did not completely restore the accumulation. The antimicrobial efficacy of ZnO-MNPs was investigated and showed potential results against common pathogens, including *Bacillus subtilis* (29.05 ± 0.76), *Bacillus meurellus* (27.05 ± 0.5), *Acetobacter rhizospherensis* (23.36 ± 0.5), and *Escherichia coli* (25.86 ± 0.80), while antifungal activity was relatively lower. Moreover, the 2,2-diphenyl-1-picrylhydrazyl (DPPH) assay showed that ZnO-MNPs are strong antioxidant agents. Overall, these findings highlight the effectiveness of myco-synthesized ZnO-NPs in combating pathogenic diseases, their promising role in cancer therapy, and their potential as a biomaterial option for future therapeutic applications.

## 1. Introduction

Nanotechnology, or manipulating matter at the atomic and molecular level, has the ability to transform industries, technology, and medicine, making it a major player in a wide range of areas [1]. In medicine, it allows drugs to be administered accurately, increases the effectiveness of treatments while reducing side effects, and facilitates the use of sophisticated diagnostic tools to detect early disease [1,2]. Regarding these special properties, it has been named a “wonder of modern medicine” [3]. Nanotechnology in electronics is driving the development of devices that are faster, smaller, and more efficient, opening the door to breakthroughs such as quantum computing and flexible displays. Using nanomaterials and nano-filters, it provides environmentally friendly solutions to reduce pollution, clean energy, and water purification [4,5,6].

The synthesis of nanoparticles (NP) occurs using various chemical processes that are unsafe and have been identified as a major source of chemical pollution in the ecosystem, disrupting the natural habitat. It is therefore time to introduce new environmental methods that are environmentally friendly, cost-effective, and yield-rich to tackle the burden of this environmental degradation [7,8,9]. Biological or green synthesis of nanoparticles is an environmentally friendly method that allows the size and shape of the synthesized nanoparticles to be controlled by various microbes without the use of expensive and dangerous solvents [10,11,12]. Various microbes play promising roles in biological processes, but mushrooms, which belong to a richly diverse group of fungi, are considered more effective candidates due to their natural mechanism of secreting enzymes, proteins, amino acids, polysaccharides, and vitamins for the synthesis of nanoparticles from metal salts [13,14]. Without the need for parameters such as temperature or other hazardous chemicals, mycogenic synthesis of NPs is the simplest and most cost-effective method for producing stable nanoparticles [15,16,17,18]. As a result, many researchers have started to prefer biological approaches, particularly myco-synthesis, over the chemosynthetic approach [19].

Several nanoscale metal oxides have been prepared and used in various fields, including CuO, TiO_2_, and ZnO [20]. Among them, ZnO nanoparticles have attracted special attention in the biomedical field due to their ease of production, non-toxicity, and safety [21]. The US Food and Drug Administration approved the use of ZnO nanoparticles as safe metal oxides [20]. Many physiological processes, such as immune system, antioxidant function, growth, skeletal development, skin growth, appetite, wound healing, and reproductive system, depend on zinc, which is a necessary nutritional supplement. These processes also support the use of zinc in drug delivery to maintain health [22]. ZnO NPs showed toxicity towards many hazardous diseases, including cancer. Cancer is the second most common cause of death worldwide, which is 13 percent of all deaths, with an estimated 7.6 million deaths per year [23]. By 2030, the number of deaths from cancer will probably increase by 13 million, and the prevalence of cancer is expected to deteriorate soon. Lung cancer, the most common cancer and the leading cause of cancer-related deaths worldwide, represents around 15 percent of all new cancer diagnoses [24]. Numerous harmful substances infiltrate the body and cause fatal diseases. Carbon tetrachloride (CCl_4_) is also included in this list; its vapors have the potential to degenerate the liver and kidneys and suppress central nervous system activity. Evidence of carcinogenicity in experimental animals suggests that carbon tetrachloride is likely to cause cancer in humans, which was the main concern that focused on the need to examine the hepatotoxicity caused by CCl_4_ and potential preventive measures [25,26]. Zinc oxide nanoparticles are versatile in nature; they can fight against different resistant microbe strains and appear as strong antimicrobial agents [27]. The synthesis of ZnO NPs is the main focus of rapidly developing bio-nanotechnology “nanomedicine” in the pharmaceutical industry due to their healing, catalytic, anti-inflammatory, antibacterial, anti-cancer, and antidiabetic properties [28,29].

In this study, we demonstrated green myco-synthesis of ZnO-MNPs from extract from a mushroom (*Cortinarius* sp.). The main objective of this study was to determine the therapeutic effects of the mushroom extracts and their ZnO-MNPs by looking at the effects of these substances on anti-cancer, antimicrobial, and antioxidant properties.

## 2. Materials and Methods

### 2.1. Sample Collection and Processing

**Figure 1:**
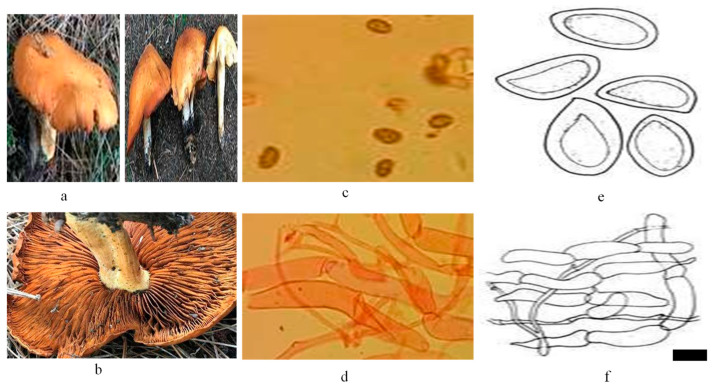
(**a**,**b**) Morphological representation of fresh basidiomata of *Cortinarius* sp. (**c**) Anatomical representation of basidiospores by LM (**d**). Anatomical representation of pileipellis by LM. (**e**) Illustrations of basidiospores, (**f**) Illustrations of pileipellis. Scale Bar: a = 1.31 cm, b = 8.3 µm, c = 8.2 µm, d = 29.65 µm, e = 4.90 µm, f = 35.52 µm.

Numerous edible and non-edible species of wild mushrooms were collected from Kumrat Valley in KPK, Pakistan. Specific codes were applied to the specimens, and images of the newly arrived specimens in their natural environment were captured using a Canon EOS 3000D (Rebel T100) DSLR camera with an 18–55 mm DC III lens Melville, NY, USA to facilitate feature detection and nanoparticle myco-synthesis. The collected samples were handled, stored, and transported according to appropriate guidelines. Furthermore, the *Cortinarius* sp. was identified based on morpho-anatomical characteristics (Figure 1) and investigated for the synthesis of zinc oxide (ZnO) myco-nanoparticles and screened for therapeutic potential. 

### 2.2. Chemicals

The Department of Botany, Government College University, Lahore, Pakistan, provided the standard zinc chloride (ZnCl_2_), carbon tetrachloride (CCl_4_), ethanol, and 10 percent formalin, among other chemicals and reagents used in this study.

### 2.3. Preparation of Mushroom Extract and Myco-Synthesis of ZnO Myco-Nanoparticles

The sample of mushrooms was thoroughly washed 3 times with double distilled water (ddH_2_O) to remove dust and debris from the samples. The samples were then surgically cut into tiny pieces and left in the shade for 3–4 days to become dry. Furthermore, the dried sample materials were ground into fine powder. A further 10 g of the mushroom powder sample were weighed using the electronic balance (DJ-E 100A), mixed with 500 mL of deionized water, and heated at 50 °C for 30 min. The extract was filtered using Whatman No. 1 filter paper with a pore size of 11 μm and was utilized for the synthesis of zinc oxide myco-nanoparticles and stored at 5 °C for additional procedures. In a beaker, 30 mL of aqueous mushroom extract was added to 70 mL of 0.02 M ZnCl_2_ solution with constant stirring. The mixture was dissolved, and then NaOH solution (2 M) was added after every five minutes with constant stirring until the pH reached up to 7.0. The addition of NaOH solution resulted in the formation of a cloudy solution after 2 h. Then, the solution was centrifuged at 4000 rpm for 10–15 min (centrifuge: Model: Eppendorf 5424 R, Germany, with 24 × 2 mL A-45-24-11 Rotor). The light brown pellet was collected and further washed twice with ddH_2_O and once with ethanol to remove any remaining impurities. After that solution (extract + ZnO-MNPs) was poured into Petri dishes and dried overnight in the oven to make a powder of nanocrystals to use for additional processing. Myogenic ZnO-MNPs nanoparticles were purified and turned pure white from light brown after calcination in an oven at 700 °C for 2 h. The ZnO-MNPs nanoparticles were then stored at room temperature for further structural analysis and biological screening.

### 2.4. Structural Analysis of ZnO MNPs

The synthesized ZnO-MNPs were characterized using various methods, such as the UV-Vis spectrometer (Perkin Elmer; Lambda 25) (PerkinElmer, Inc., Waltham, MA, USA) used to analyze the optical properties of the ZnO-MNPs. An XRD (model D8 ADVANCE, Bruker Corporation, Billerica, MA, USA) was used to record the XRD pattern. The model provided information on translational symmetry, the size of metallic ZnO-MNPs, and phase identification. The functional groups attached to the synthesized ZnO-MNP surface were examined by Fourier transmission infrared spectroscopy (FTIR) (Perkin Elmer Paragon 500).

### 2.5. Biomedical Applications of ZnO-MNPs

#### 2.5.1. Evaluation of Anti-Cancer Potential of ZnO-MNPs

##### Ethical Approval

The animal experiment followed both local and international rules and regulations. The rule recognized worldwide by Dutch law (internationally), i.e., Wet op de dieaproeven (Article 9), was followed as mentioned in a previous study [30]. The Institutional Review Board of Government College University, Lahore, gave its approval for this study.

##### Experimental Animals and Dose Preparation

**Figure 2:**
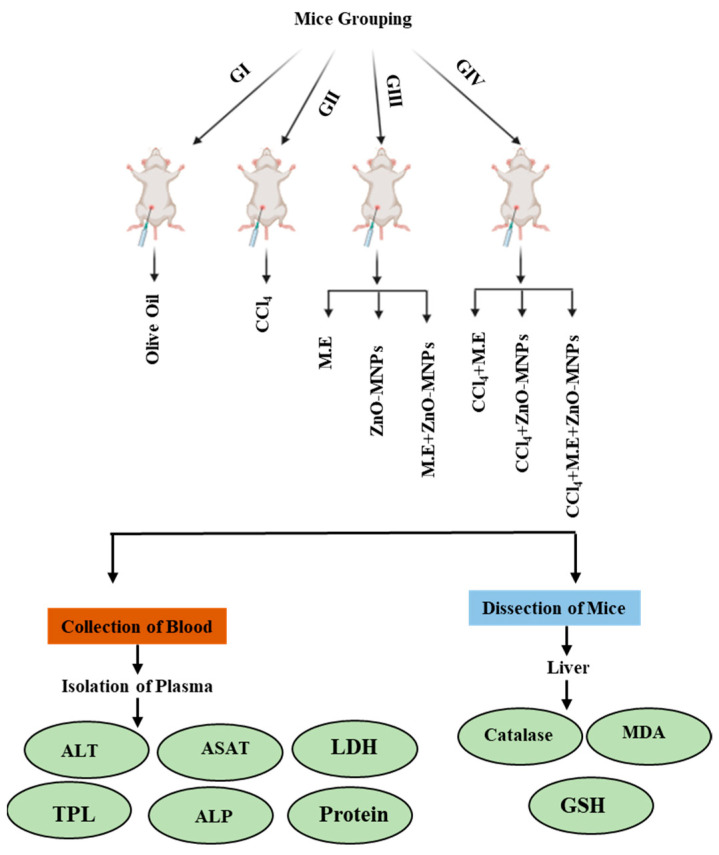
Schematic overview of key parameters assessed during blood collection and post-dissection tissue analysis in the experimental study.

For this study, 40 Balb C mice (*Mus musculus*) with an average weight of 25 g were purchased from the University of Veterinary and Animal Sciences, Lahore, Pakistan. Mice were maintained in an animal house at 20 °C ± 2 °C with 12-h light and dark cycles at the Department of Zoology, Government College University, Lahore. They were regularly provided with drinking water and standard mice pellets as needed. The mice were divided into four groups, namely (I), (II), (III), and (IV). Group (I) contained 5 mice that were given intraperitoneal olive oil treated as a negative control. Group (II) contained 5 mice in which hepatotoxicity was caused by giving intraperitoneal CCl_4_ (0.4 ml/kg) dissolved in an equal volume (*v*/*v*) of olive oil as a positive control. Group (III), with 15 mice, was divided into three sub-groups named (i) M.E, (ii) ZnO-MNPs, and (iii) M.E. + ZnO-MNPs, fed by mushroom extract, ZnO-MNPs, and doses of both mushroom extract and ZnO-MNPs, respectively, with a concentration of 3 mg/kg, 3 times a week for 35 days. Group (IV) had 15 mice. First, they were given CCl_4_ (0.4 ml/kg) dissolved in olive oil (*v*/*v*) for 2 weeks on alternate days to cause liver damage. Then, the mice were split into 3 different groups for their treatment: (i) treated with mushroom extract, (ii) treated with ZnO-MNPs, and (iii) treated with mushroom extract and ZnO-MNPs. Each treatment was given at a concentration of 3 mg/kg, 4 times a week for 21 days as shown in (Figure 2).

##### Biochemical Analysis

Mice after 35 days were fasted overnight, then euthanized, and blood samples were collected in ethylenediaminetetraacetic acid (EDTA) tubes containing approximately 1.8 mg of K_2_EDTA per milliliter of blood. Blood samples were used to measure liver biochemical parameters such as total bilirubin, protein, catalase, melanin dialdehyde (MDA), glutathione (GSH), alanine aminotransferase (ALT), aspartate aminotransferase (ASAT), alkaline phosphatase (ALP), and lactate dehydrogenase (LDH). Biochemical parameters such as alanine transaminase (ALT) were performed according to the previous method by Bowers [31], and aspartate transaminase (AST) was estimated by using the method of Reitman and Frankel [32]. The total bilirubin level (TBL) was determined using the modified dimethyl sulfoxide (DMSO) method [33]. Lactate dehydrogenase (LDH) was measured according to technique [34]. Level of total protein was estimated using the previously described method [35]. Catalase activity was assessed by a previously described standard protocol [36]. GSH was measured according to the standard protocol [37], and MDA was measured according to the previously described method [38].

#### 2.5.2. Evaluation of Antimicrobial Potential of ZnO-MNPs

Gram-positive *Bacillus meurellus* and *Bacillus subtilis,* as well as Gram-negative *Escherichia coli* and *Acetobacter rhizospherensis* were tested for their antibacterial potential using three different test samples: M.E. (mushroom extract), ZnO-MNPs (zinc oxide myco-nanoparticles), and M.E. + ZnO-MNPs (mushroom extract + zinc oxide myco-nanoparticles). Antifungal activity was evaluated against the phytopathogenic fungi *Aspergillus flavus* and *Mucor mucedo*. The Department of Biotechnology, GC University, Lahore, Pakistan, provided all microbial strains used in this study, which were then screened using the disk diffusion method [39] with minor modifications.

##### Antibacterial Activity

The bacterial sample stock cultures *(Bacillus subtilis*, *Bacillus meurellus*, *Acetobacter rhizospherensis,* and *Escherichia coli)* were inoculated in 50 µL of LB medium and further cultured at 37 °C for 24 h. Bacterial suspensions were adjusted to a 0.5 McFarland standard (1.5 × 10^⁸^ CFU/mL). Furthermore, 100 µL of each bacterial strain was dropped onto the solidified medium and evenly distributed using a sterile glass spreader. Sterile antibiotic disk (6 mm diameter, Oxoid, Thermo Fisher Scientific, Hampshire, UK) was separately loaded with test samples M.E., ZnO-MNPs, and M.E. + ZnO-MNPs in different concentrations of 200, 300, 400, and 500 µg/mL and placed on Petri dishes. Streptomycin was used as a control measure. The cultured agar plates were further incubated at 37 °C for 24–48 h. After that, the zone of inhibition was recorded in millimeters (mm). For positive control, a broad-spectrum antibiotic was utilized. The experiment was repeated three times and yielded similar results.

##### Antifungal Activity

The PDA medium was autoclaved at 121 °C for 20 min and then poured into sterilized Petri dishes under a laminar airflow hood (ENVAIR). Previously preserved fungal strains were rejuvenated by inoculating 50 µL of PDA medium and incubating at 37 °C for 24 h. Finally, to ensure uniform distribution, 100 µL of each fungal strain was added and spread over the solidified PDA medium using a sterilized glass spreader. Using test samples of mushroom extract, ZnO-MNPs, and mushroom extract + ZnO-MNPs at different concentrations of 200, 300, 400, and 500 μg/mL, each sterile antibiotic disk (6 mm diameter, Oxoid, Thermo Fisher Scientific, Hampshire, UK) was individually loaded and placed on Petri dishes. Streptomycin was used as a positive control, and Petri dishes were further incubated at 37 °C for 24–48 h. After that, the zone of inhibition was recorded in millimeters (mm). The experiment was repeated three times and yielded similar results.

#### 2.5.3. Evaluation of Antioxidant Potential of ZnO-MNPs

The 1,1,-diphenyl-2-picrylhydrazyl assay was used to measure the free radical scavenging activity, or antioxidant activity, of three different test samples: M.E. (mushroom extract), ZnO-MNPs (zinc oxide myco--nanoparticles), and M.E. + ZnO-MNPs (mushroom extract plus zinc oxide myco-nanoparticles). Three milliliters of each of the mentioned samples at different concentrations (200, 300, 400, 500 µg/mL) were placed in glass vials and covered with aluminum foil. In order for the reaction to proceed, the mixture was shaken vigorously to ensure that the two solutions mixed evenly and then left at room temperature for half an hour. The absorption value at 517 nm was then measured using a spectrophotometer. Antioxidant activity was higher when absorption values were lower. The control substance was ascorbic acid. The experiment was repeated three times and yielded similar results.

DPPH radical scavenging effect% = [(A0 − A1/A0)] × 100

where:

A0 = Absorbance of the control.

A1 = Absorbance in the presence of a sample.

### 2.6. Statistical Analysis

All experimental data were analyzed using GraphPad Prism version 9 (GraphPad Software, San Diego, CA, USA). All values are presented with mean ± SD as indicated. Data points are plotted onto the graphs, and the number of samples is indicated in the corresponding figure legends. A *p-*value < 0.05 was considered to indicate statistical significance. The graphs were generated with the GraphPad Prism 9 software.

## 3. Results

### 3.1. Characterization

#### 3.1.1. XRD (X-Ray Diffraction) Analysis of ZnO-MNPs

**Figure 3:**
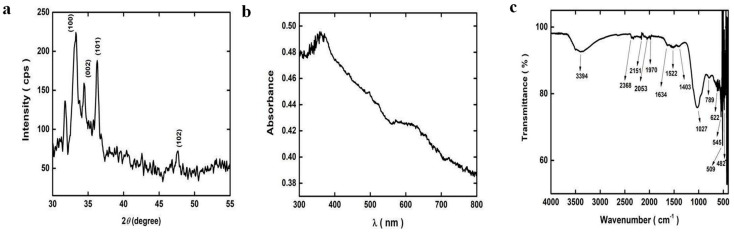
(**a**) XRD pattern, (**b**) UV-vis spectra, and (**c**) FTIR spectra of ZnO-MNPs.

The Cu-Kα radiation with a wavelength of 0.15406 nm was used as a source to obtain the XRD pattern of ZnO-MNPs. The diffraction pattern was recorded at room temperature (RT) with a scanning speed of 4° per minute in the 2θ range from 20 °C to 80 °C. Diffraction peaks for planes (100), (002), (101), and (102) in the sample show their polycrystalline character. According to JCPDS card number 01-089-1397, all diffraction peaks showed a hexagonal wurtzite structure. The width showed that the crystallites of the nanoparticles were small. The ideal orientation of the sample was visible along the 102 peaks in the XRD patterns (Figure 3a).

#### 3.1.2. Ultraviolet Visible Spectroscopy Analysis of ZnO-MNPs

UV-visible spectroscopy was used to determine the spectrum of ZnO-MNPs in suspension. At a scanning speed of 480 nm/min, the ultraviolet–visible energy range of the electromagnetic spectrum was 1.5–6.2 eV, or the wavelength range was 300–800 nm. The absorption peak of the ZnO-MNPs was discovered at around 378 nm (Figure 3b).

#### 3.1.3. FTIR Analysis of ZnO-MNPs

FTIR was used to identify the functional groups of the synthesized ZnO-MNPs. FTIR spectra in the range of 500–4000 cm^−1^ were recorded in the solid phase using the KBr pellet technique. This absorption peak at 3394 cm^−1^ is attributed to the stretching vibrations of the O-H groups of phenol and alcohol. It is possible to correlate the stretching vibration of the carbonyl groups with the band at 1634 cm^−1^. The two prominent peaks at 1522 cm^−1^ and 1403 cm^−1^ were signs of nitro groups. The peak at 1027 cm^−1^ corresponds to the C-N stretch of an amino acid. The characteristic Zn-O peaks corresponding to the stretching mode of the Zn-O bond were between 789 cm^−1^ and 545 cm^−1^. The characteristic Zn-O bond absorption peak at 545 cm^−1^ was observed (Figure 3c).

### 3.2. Biomedical Applications of ZnO-MNPs

#### 3.2.1. Anti-Cancer Activity (Effects on Biochemical Components)

##### Effect on ALAT

**Figure 4:**
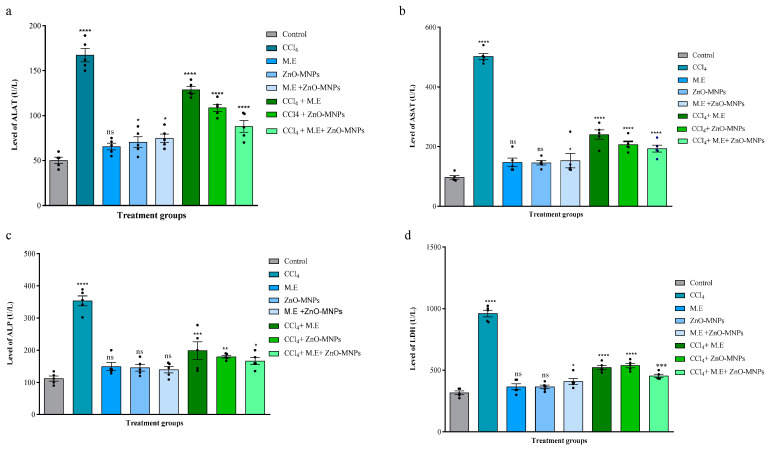
Anti-cancer activity effects on biochemical components (**a**) Analysis of alanine transaminase (ALAT). (**b**) Analysis of aspartate aminotransferase (ASAT) (**c**) Analysis of alkaline phosphatase (ALP) (**d**) Analysis of lactate dehydrogenase (LDH). Key: CCl_4_ represents carbon tetrachloride group; M.E. and ZnO-MNPs stand for mushroom extract and zinc oxide myco-nanoparticles with concentration of 3 mg/kg; CCl_4_ + M.E, CCl_4_ + ZnO-MNPs, and CCl_4_ + M.E + ZnO-MNPs represent the treatment groups of CCl_4_-injected mice after 14 days of using mushroom extract, ZnO-MNPs, and mushroom extract + ZnO-MNPs. Each bar represents the mean value of five replicates and SEM. Asterisks indicate significant differences by Student *t*-test. * *p* < 0.05; ** *p* < 0.01; *** *p* < 0.001; **** *p* < 0.0001; ns non-significant.

The results suggested that the ALAT level (167.2 ± 6.1 U/L) was increased significantly when CCl_4_ (0.4 mL/kg body weight) was administered intraperitoneally to cause hepatotoxicity in mice. This was in contrast to the following values: (Control: 50.2 ± 2.9 U/L; Mushroom extract: 65.6 ± 3.1 U/L; ZnO-MNPs: 70.4 ± 5.1 U/L; Mushroom extract + ZnO-MNPs: 74.8 ± 3.9 U/L). However, the hepatotoxic mice group showed a highly significant decrease in its level when treated with CCl_4_ + M.E. (128.6 ± 3.08 U/L), CCl_4_ + ZnO MNPs (108.6 ± 3.4 U/L), and CCl_4_ + M.E. + ZnO MNPs (88 ± 5.4 U/L) compared to CCl_4_. The results revealed that the treatment group of ZnO-MNPs + ME reduced the higher level of ALAT caused by CCl_4_ as shown in Figure 4a.

##### Effect on ASAT

When CCl_4_ (0.4 mL/kg body weight) was administered intraperitoneally, we found that the ASAT level (502 ± 8.8 U/L) was significantly higher as compared to values of other groups: the control group (90.2 ± 2.1 U/L), the mushroom extract group (132.8 ± 4.8 U/L), the ZnO-MNPs group (140.2 ± 3.6 U/L), and the mushroom extract + ZnO-MNPs group (128.8 ± 2.6 U/L). However, CCl_4_-treated hepatotoxic mice were treated with different treatment groups, and the mushroom extract group, the ZnO-MNPs group, and the mushroom + ZnO-MNPs group (3 mg/kg body weight) showed a significant decrease in ASAT levels (CCl_4_ + M.E.: 240.2 ± 13.13 U/L; CCl_4_ + ZnO-MNPs: 206.6 ± 9.08 U/L; CCl_4_ + M.E. + ZnO-MNPs: 193 ± 9.8 U/L). Among all, results revealed the treatment group of ZnO-MNPs + ME reduced the higher level of ASAT caused by CCl_4_ as shown in Figure 4b.

##### Effect on ALP

The results showed a significant increase in ALP levels (353.4 ± 12 U/L) when CCl_4_ (0.4 mL/kg body weight) was administered intraperitoneally as compared to the control group (111.6 ± 6.5 U/L), the mushroom extract group (134.8 ± 2.3 U/L), the ZnO-MNPs group (145.4 ± 8.9 U/L), and the mushroom extract + ZnO-MNPs group (139.2 ± 8.3 U/L). The hepatotoxic mice showed a significant decrease in ALP levels after administration of different treatment groups such as the mushroom extract group, the ZnO-MNPs group, and the mushroom + ZnO-MNPs group (3 mg/kg body weight) (CCl_4_ + M.E.: 199 ± 22 U/L; CCl_4_ + ZnO-MNPs: 179.8 ± 3.5 U/L; CCl_4_ + M.E. + ZnO-MNPs: 166.2 ± 9.1 U/L). Results revealed that the treatment group of ZnO-MNPs + ME reduced the higher level of ALP caused by CCl_4_, as shown in Figure 4c.

##### Effect on LDH

Our results revealed intraperitoneal injection of CCl_4_ (0.4 mL/kg body weight) significantly increased LDH level (960.4 ± 23 U/L) compared to the following conditions: control: 306.4 ± 10 U/L; mushroom extract: 365.2 ± 20.8 U/L; ZnO-MNPs: 362.8 ± 11.10 U/L; and mushroom extract + ZnO-MNPs: 387.2 ± 6.9 U/L. Its content was significantly lower when treated with different treatment groups, such as the mushroom extract group, the ZnO-MNPs group, and the mushroom + ZnO-MNPs group (CCl_4_ + M.E.: 522.4 ± 14 U/L; CCl_4_ + ZnO MNPs: 538.4 ± 14 U/L; and CCl_4_ + M.E. + ZnO-MNPs: 538.4 ± 14 U/L). Results revealed that the treatment group of ZnO-MNPs + ME reduced the higher level of LDH caused by CCl_4_, as shown in Figure 4d.

##### Effect on GSH

**Figure 5:**
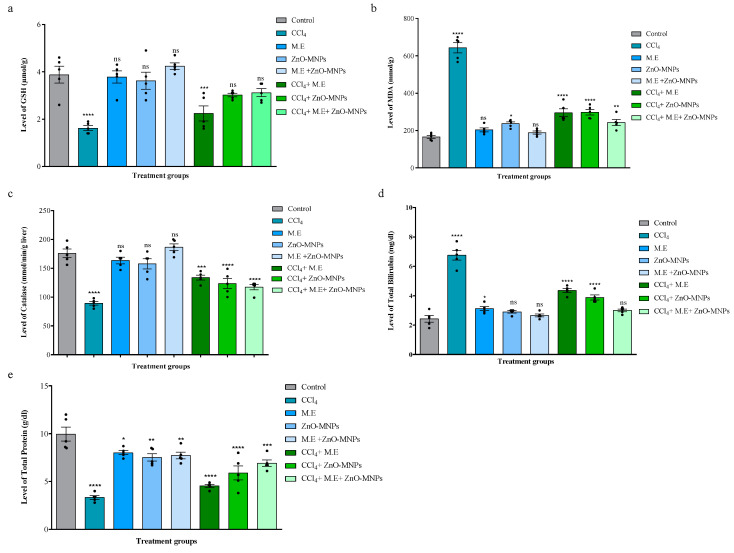
Anti-cancer activity effects on biochemical components. (**a**) Analysis of glutathione (GSH). (**b**) Analysis of melanin dialdehyde (MDH). (**c**) Analysis of catalase. (**d**) Analysis of total bilirubin. (**e**) Analysis of total protein. Key: CCl_4_ represents the carbon tetrachloride group; M.E. and ZnO-MNPs stands for mushroom extract and zinc oxide myco-nanoparticles with concentration of 3 mg/kg while CCl_4_ + M.E., CCl_4_ + ZnO-MNPs, and CCl_4_ + M.E + ZnO-MNPs represent the treatment groups of CCl_4_-injected mice after 14 days of using mushroom extract, ZnO MNPs, and mushroom extract + ZnO MNPs. Each bar represents the mean value of five replicates and SEM. Asterisks indicate significant differences by Student *t*-test. * *p* < 0.05; ** *p* < 0.01; *** *p* < 0.001; **** *p* < 0.0001; ns non-significant.

The results revealed that the GSH level (1.62 ± 0.08 µmol/g) was significantly lower after intraperitoneal administration of CCl_4_ (0.4 mL/kg by body weight) compared to values of the control group (3.88 ± 0.2 µmol/g), mushroom extract group (3.78 ± 0.21 µmol/g), ZnO-MNPs group (3.78 ± 0.3 µmol/g), and mushroom extract + ZnO-MNPs group (4.24 ± 0.11 µmol/g). GSH levels in hepatotoxic mice were significantly restored in different treatment groups, such as the mushroom extract group, the ZnO-MNPs group, and the mushroom + ZnO-MNPs group (CCl_4_ + M.E.: 2.24 ± 0.2 µmol/g; CCl_4_ + ZnO-MNPs: 3.02 ± 0.06 µmol/g; CCl_4_ + M.E. + ZnO-MNPs: 3.12 ± 0.14 µmol/g), as shown in Figure 5a.

##### Effect on MDA

The results of this study showed that intraperitoneal addition of CCl_4_ (0.4 mL/kg body weight) resulted in a significant increase in MDA levels (643.4 ± 22 mmol/g) compared to the control group (166.2 ± 6.9 mmol/g), the mushroom extract group (203.8 ± 8.8 mmol/g), the ZnO-MNPs group (237.4 ± 7.4 mmol/g), and the mushroom extract + ZnO-MNPs group (187 ± 6.1 mmol/g). Treatment groups with hepatotoxicity in mice were treated with different treatment groups, resulting in significant reductions in MDA, ZnO-MNPs, and mushroom extract + ZnO-MNPs (3 mg/kg body weight), resulting in a significant reduction in MDA level (CCl_4_ + M.E.: 295.6 ± 22 mmol/g; CCl_4_ + ZnO-MNPs: 298.4 ± 12 mmol/g; CCl_4_ + M.E. + ZnO-MNPs: 243.4 ± 13 mmol/g) as compared to CCl_4_, as shown in Figure 5b.

##### Effect on Catalase

The results showed that intraperitoneal injection of CCl_4_ (0.4 mL/kg body weight) significantly reduced catalase levels (89.2 ± 2.6 mmol/min/g) in the hepatotoxic mice group as compared to the control: 175.8 ± 6.2 mmol/min/g; mushroom extract: 163.4 ± 4.7 mmol/min/g; ZnO-MNPs: 157.6 ± 7.3 mmol/min/g; and mushroom extract plus ZnO-MNPs: 186.4 ± 4.9 mmol/min/min/g. The hepatotoxic mice groups were treated with different treatment groups by administration of mushroom extract, ZnO-MNPs, and mushroom extract + ZnO-MNPs (3 mg/kg body weight). The results revealed that the level of catalase significantly started to increase in treatment groups (CCl_4_ + M.E: 133.8 ± 3.5 mmol/min/g; CCl_4_ + ZnO-MNPs: 123.6 ± 7.4 mmol/min/g; CCl_4_ + M.E + ZnO MNPs: 117.2 ± 3.8 mmol/min/g), as shown in Figure 5c.

##### Effect on Total Bilirubin

The results showed that intraperitoneal administration of CCl_4_ (0.4 mL/kg by body weight) increased total bilirubin levels (6.76 ± 0.2 mg/dL) as compared to the values of the control group (2.42 ± 0.18 mg/dL), mushroom extract group (3.12 ± 0.11 mg/dL), ZnO-MNPs (2.9 ± 0.06 mg/dL), and mushroom extract + ZnO-MNPs (2.7 ± 0.06 mg/dL). However, when different treatment groups, such as mushroom extract, ZnO-MNPs, and mushroom extract + ZnO-MNPs (3 mg/kg body weight), were administered intraperitoneally to hepatotoxic mice groups, the total bilirubin level started to decrease as follows: CCl_4_ + M.E.: 4.36 ± 0.1 mg/dL; CCl_4_ + ZnO-MNPs: 3.88 ± 0.1 mg/dL; and CCl_4_ + M.E. + ZnO-MNPs: 2.66 ± 0.08 mg/dL (Figure 5d).

##### Effect on Total Protein

Our data revealed that the total protein content (3.24 ± 0.1 g/dL) after intraperitoneal administration of CCl_4_ was significantly reduced (0.4 mL/kg body weight) as compared to the control (9.96 ± 0.6 g/dL), mushroom extract (8.02 ± 0.18 g/dL), ZnO-MNPs (7.52 ± 0.3 g/dL), and mushroom extract + ZnO-MNPs (7.72 ± 0.29 g/dL). The hepatotoxic mice groups were treated with different treatment groups by giving doses of mushroom extract, ZnO-MNPs, and mushroom extract + ZnO-MNPs (3 mg/kg body weight). The results revealed that the total protein level started to recover (CCl_4_ + M.E.: 4.54 ± 0.12 g/dL; CCl_4_ + ZnO-MNPs: 5.9 ± 0.6 g/dL; CCl_4_ + M.E. + ZnO-MNPs: 6.92 ± 0.2 g/dL), as shown in Figure 5e.

### 3.3. Antimicrobial assays

#### 3.3.1. Antibacterial Activity

**Figure 6:**
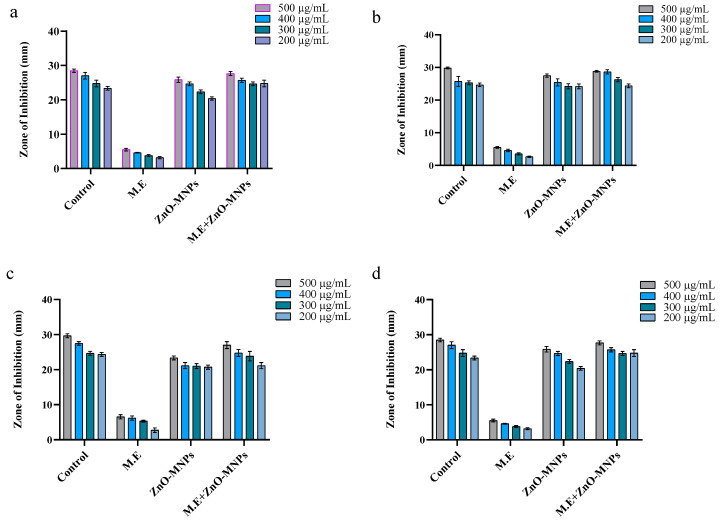
Graphical representation of antibacterial activity of different samples at varying concentrations against (**a**) *Bacillus subtilis*, (**b**) *Bacillus meurellus*, (**c**) *Acetobacter rhizospherensis,* and (**d**) *Escherichia coli*. Error bars represent means ± SD of three biological replicates.

**Figure 7:**
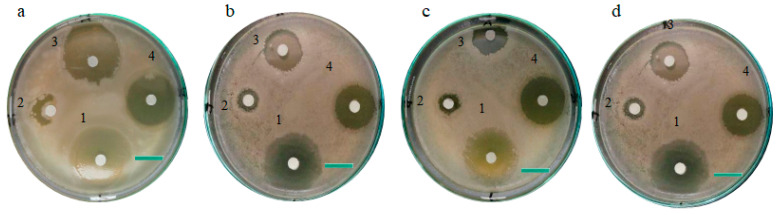
Zone of inhibition showing antibacterial activity of different samples (1. Control, 2. M.E., 3. ZnO-MNPs, and 4. ZnO-MNPs + M.E.) at 500 µg/mL concentrations against (**a**) *Bacillus subtilis*, (**b**) *Bacillus meurellus*, (**c**) *Acetobacter rhizospherensis,* and (**d**) *Escherichia coli*. Scale Bar: 1cm.

As ZnO-MNPs are toxic for pathogenic microorganisms, they are of great importance. Synthetic ZnO-MNPs derived from the *Cortinarius* sp. were used to evaluate the antibacterial activity against both Gram (−) and Gram (+) bacteria. At four different concentrations (200, 300, 400, and 500 µg/mL), the diameter of the clear zone formed by ZnO-MNPs, M.E., and ZnO-MNPs plus M.E. was used to determine and confirm the inhibitory actions of all samples. ZnO-MNPs showed exceptional toxicity against both Gram-positive and Gram-negative bacteria in all test samples. However, the inhibition zone of the Gram-positive bacteria was broader and significantly clearer. Therefore, ZnO-MNPs showed greater antibacterial potential against Gram-positive bacteria compared to Gram-negative bacteria. All testing samples (i.e., ZnO-MNPs, ii. M.E., iii. ZnO-MNPs plus M.E.) showed significant results at the concentration of 500 µg/mL, and the diameter of the clear zone formed by ZnO-MNPs was recorded as 29.05 ± 0.76, 27.05 ± 0.5, 23.36 ± 0.5, and 25.86 ± 0.80 mm against *Bacillus subtilis*, *Bacillus meurellus*, *Acetobacter rhizospherensis,* and *Escherichia coli* (Figure 6a–d and Figure 7a–d). 

#### 3.3.2. Antifungal Activity

**Figure 8:**
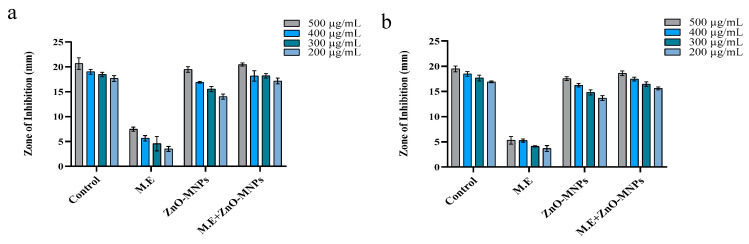
Antifungal activity. The activity of different samples at different concentrations against (**a**) *Mucor mucedo* and (**b**) *Aspergillus flavus*.

ZnO-MNPs, M.E., and ZnO-MNPs plus M.E. were tested for their antifungal properties against two different strains of pathogenic fungi in different concentrations of 200, 300, 400, and 500 µg/mL. All test samples were toxic in all concentrations for fungi, but myco-synthetic zinc oxide nanoparticles and mushroom extract samples with 500 µg/mL led to amazing results in the form of a clear inhibition zone in mm. The toxic effect of ZnO-MNPs on fungal strains led to clear zones with diameters of 19.46 ± 0.55 mm for *Mucor mucedo* and 17.53 ± 0.37 mm for *Aspergillus flavus* (Figure 8a,b). Literature evidence supports the antifungal potential of bio-synthesized NPs against *Sclerotium sclerotia*, *Alternaria alternata*, *Aspergillus terreus*, *Aspergillus niger*, *Alternaria solani*, *Fusarium solani,* and *Fusarium oxysporum* [40,41,42,43].

#### 3.3.3. Antioxidant Activity

**Figure 9:**
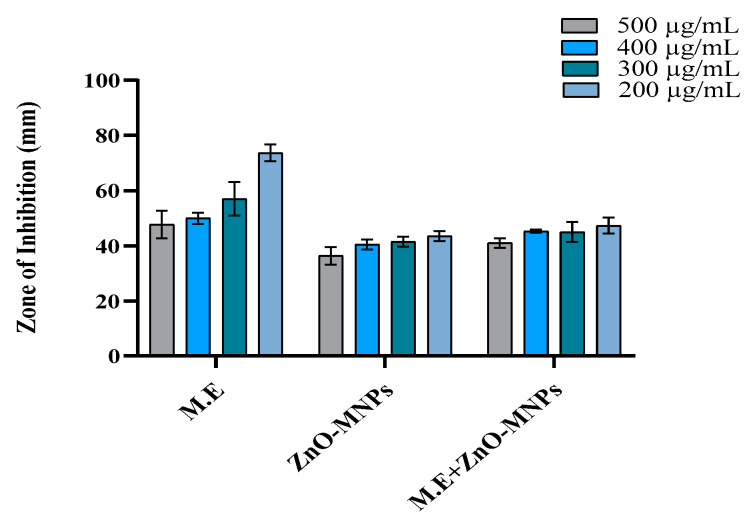
Antioxidant activity. DPPH radical scavenging activity of different testing samples at different concentrations.

ZnO-MNPs, M.E., and ZnO-MNPs plus M.E, were demonstrated to have radical scavenging activity (Figure 9). The antioxidant activity was measured using test samples at different concentrations (200, 300, 400, and 500 µg/mL). When ZnO-MNPs, ZnO-MNPs plus M.E., and M.E. were present at a minimum concentration of 200 µg/mL, their scavenging potential was 45 percent, 49 percent, and 73 percent, respectively. At the maximum concentration of 500 µg/mL, the results were 40.1 percent, 43.5 percent and 65 percent. According to the results, ZnO-MNPs have higher antioxidant potential than other samples (Figure 9).

## 4. Discussion

*Cortinarius* is the largest genus of fungi that has worldwide distribution and consists of about 3000 species around the world. *Cortinarius* sp. is rich in proteins, polysaccharides, vitamins, and amino acids [44]. This species is used as a major reducing, stabilizing, and capping agent in the synthesis of ZnO-MNPs from the metal salts of ZnCl_2_ and is considered a suitable candidate for green synthesis of nanoparticles among other available microbes. A variety of experiments have been conducted on the utilization of fungal extract for the bio-synthesis of ZnO-NPs; among them, the researcher group of Mohamed used *Penicillium chrysogenum* [1], and some other groups used *Pleurotus platypus* [43,45,46]; *Ganoderma applanatum* and Volvariella *volvacea* [43]; and *Schizophyllum commune* and *Tricholoma matsutake* [47] for the green synthesis of unique nanoparticles.

In Figure 3a, the ZnO-MNPs XRD patterns are displayed. Diffraction peaks for planes (100), (002), (101), and (102) were typically observed at 2θ. ZnO-MNPs were found to have a hexagonal crystalline structure, which was similar to the shape discovered by [48] when ZnO nanoparticles were analyzed using fungi species. Figure 3b showed the UV-visible spectra of ZnO-MNPs by *Cortinarius* sp. revealed the highest absorption at 379 nm, which fully relates to the findings of [49], who produced ZnO nanoparticles from leaf extract of a plant and showed an absorption at 380 nm. This observation is in accordance with earlier studies on the ZnO-NPs biosynthesis, which showed the characterization peak of ZnO-NPs at 380 nm [50]. Another study revealed that ZnO-NPs made from *Tabernaemontana divaricata* had maximum absorption peaks at 376 nm [51]. FTIR analysis of ZnO-MNPs revealed unique IR bands, as seen in Figure 3c. These findings were in line with those of [48], who determined the different functional groups of ZnO NPs made from the fungal strain by FTIR analysis. Our investigation into the interpretation of the IR spectra band was also strongly supported by the previous observation [1].

According to Marcellin and Kutala [52], the liver detoxifies dangerous foreign substances that help the body’s defense system. To evaluate the anti-cancer potential of ZnO-MNPs and mushroom extract, we treated Balb C mice with CCl_4_ to induce hepatotoxicity. Several previous studies showed that CCl_4_ contributed to hepatocellular damage in experimental models [26,53,54]. According to [55], CCl_4_-induced hepatotoxicity increased bilirubin, ALAT, ALP, ASAT, LDA, and MDA levels. When comparing Balb C mice with the control group, the administration of CCl_4_ resulted in a significant increase in bilirubin, ALAT, ALP, ASAT, LDA, and MDA levels. When these hepatotoxic mice were given (i) M.E., (ii) ZnO-MNPs, or (iii) M.E. + ZnO-MNPs, the increased level of ALAT, ALP, ASAT, LDA, MDA, and bilirubin began to decline. This effectively repairs liver damage caused by oxidative stress induced by CCl_4_ (Figure 3). These results were confirmed by research [56]. They observed comparable results when extracts from *Helianthus annuus* seeds were given to mice whose hepatotoxicity was caused by carbon tetrachloride. These results also support the published data of [30]. These results also align with the findings of [57]; they pre-treated CCl_4_-induced hepatotoxicity in Balb C mice with extract of *Daucus carota*. In our experiments, we observed that giving CCl_4_ to mice reduced the antioxidant capacity of their liver, as shown by the drop in GSH levels, protein levels, and catalase levels. In contrast, ZnO-MNPs and mushroom extract significantly increased the activity of GSH and catalase. The results from [57] were consistent with these results when Swiss albino rats were administered SE-NPs for treatment of hepatotoxicity caused by CCl_4_. The potential in vivo cytotoxic effects of ZnO-MNPs warrant careful consideration, particularly given their promising biomedical applications. While our study demonstrates partial restoration of hepatotoxic damage in CCl_4_-treated mice, recent literature highlights the dose-dependent cytotoxicity of ZnO nanoparticles in various organ systems. For instance, a 2022 study by Selvinsimpson et al. [58] observed that smaller ZnO nanoparticles (≤50 nm) induced oxidative stress in hepatic tissues at concentrations > 5 mg/kg, leading to elevated MDA and reduced GSH levels, consistent with our findings. However, our administered dose (3 mg/kg) aligns with the safety thresholds reported in murine models [59], suggesting a balance between efficacy and toxicity. Notably, a recent study [60] emphasized that surface functionalization—such as the fungal-derived capping agents in our ZnO-MNPs—can mitigate cytotoxicity by reducing reactive oxygen species (ROS) generation. Further, recent work by Danas et al. [61] revealed that mycogenic ZnO nanoparticles exhibit organ-specific toxicity, with renal tissues showing higher susceptibility than hepatic tissues at equivalent doses. While our study focused on liver-specific biomarkers, future investigations should include histopathological analyses of kidneys, lungs, and spleen to assess systemic toxicity.

In our studies, it was observed that both bacterial and fungal development were inhibited by all test samples. However, more significant results were achieved with ZnO-MNPs and the combination of mushroom extract and ZnO-MNPs, which proves the toxicity of ZnO-MNPs against harmful microorganisms. The diameter of the clear zone formed by ZnO-MNPs against *Bacillus subtilis*, *Bacillus meurellus*, *Acetobacter rhizospherensis,* and *Escherichia coli*. Our results are consistent with previously published data on the antibacterial activity of ZnO-NPs from *Cenchrus setigerus*. Their results also showed potential antimicrobial activity against *Escherichia coli*, *Klebsiella pneumoniae, Staphylococcus aureus,* and *Bacillus subtilis* was observed at 500 μg/mL [62].

In addition, our results are comparable with [63]. According to their study, when ZnO-NPs derived from *Fumaria officinalis* and *Peganum harmala* showed strong antibacterial activity against *Clavibacter michiganensis* and *Staphylococcus aureus* at a concentration of 800 μg/mL.

The toxic effect of ZnO-MNPs on fungal strains resulted in clear zones with diameters of 17.53 ± 0.37 mm for *Aspergillus flavus* and 19.46 ± 0.55 mm for *Mucor mucedo* at 500 μg/mL. When zinc oxide nanoparticles form inhibitory zones (Figure 7), this indicates that the pathogens are eliminated by the nanoparticles’ biocidal action mechanism, which results in a membrane rupture with a high rate of formation of oxygen species on the surface. The results of this investigation are consistent with the findings of Dalal and Saravanan [64]. These results are also consistent with the results of [61]. When the antifungal activity of ZnO-NPs derived from *Newbouldia laevis* leaf extract was investigated at different doses against *Trichophyton rubrum*, *Aspergillus fumigatus,* and *Candida albicans*.

Dianati et al. [65] showed that the antioxidant activity of curcumin-mediated CM-ZnO-NPs was 18.06 percent when tested with DPPH at a concentration of 500 μg/mL. The DPPH activities (58.16 percent and 55.76 percent) in 1000 μg/mL ZnO NPs mediated by *Momordica charantia* and *Curcuma zedoaria* were reported by Ahsan et al. [66]. Antioxidant activities of (i) ZnO-MNPs, (ii) ZnO-MNPs with a combination of M.E., and (iii) M.E. were measured in our study using the DPPH assay at the highest concentration of 500 µg/mL. The results were 40.1 percent, 43.5 percent, and 65 percent, respectively. Another study [67] found that ZnO NPs derived from *Calendula officinalis* flower extract had an antioxidant activity of 32.37 percent at 500 μg/mL. One possible explanation for the variations in the antioxidant activity of ZnO NPs is that they are produced from different biological extracts, resulting in different sizes and therefore different specific surfaces.

## 5. Conclusions

The ZnO-MNPs in this study were synthesized using extracts from *Cortinarius sp*. The formation and composition of these nanoparticles were confirmed through characterization with Fourier-transform infrared spectroscopy, X-ray diffraction, and UV-vis spectroscopy. To evaluate hepatoprotective effects, carbon tetrachloride (CCl_4_), a potent hepatotoxic agent, was administered to Balb C mice at a controlled dose to induce significant liver cell damage. Both the mushroom extract and the biosynthesized ZnO-MNPs demonstrated effectiveness in mitigating the hepatic injury caused by CCl_4_. Additionally, the antimicrobial activity of the mushroom extracts and ZnO-MNPs was evaluated. The results revealed that ZnO-MNPs exhibit potent antimicrobial properties, showing efficacy against bacterial and fungal strains. These findings highlight the dual potential of ZnO-MNPs as promising candidates for antipathogenic drug development and hepatoprotective therapeutic applications.

## Data Availability

The original contributions presented in this study are included in the article. Further inquiries can be directed to the corresponding authors.

## Abbreviations

The following abbreviations are used in this manuscript:

ZnO-MNPs	Zinc oxide myco-nanoparticles
M.E	Mushroom extract
XRD	X-ray diffraction
FTIR	Fourier transform infrared spectroscopy
CCl_4_	Carbon tetrachloride
ALT	Alanine aminotransferase
ASAT	Aspartate aminotransferase
LDH	Lactate dehydrogenase
MDA	Melanin dialdehyde
